# The interplay of inflammation and remyelination: rethinking MS treatment with a focus on oligodendrocyte progenitor cells

**DOI:** 10.1186/s13024-024-00742-8

**Published:** 2024-07-12

**Authors:** Omri Zveik, Ariel Rechtman, Tal Ganz, Adi Vaknin-Dembinsky

**Affiliations:** 1 Faculty of Medicine, Hebrew University of Jerusalem, Jerusalem, 91120 Israel; 2 The Department of Neurology and Laboratory of Neuroimmunology, The Agnes-Ginges Center for Human Neurogenetics, Hadassah-Hebrew University Medical Center, Ein–Kerem P.O.B. 12000, Jerusalem, 91120 Israel

**Keywords:** Demyelination, Immune-modulation, Immune-suppression, Multiple sclerosis, Myelin, Neurodegeneration, Neuroinflammation, Oligodendrocyte progenitor cell, Regeneration, Remyelination

## Abstract

**Background:**

Multiple sclerosis (MS) therapeutic goals have traditionally been dichotomized into two distinct avenues: immune-modulatory-centric interventions and pro-regenerative strategies. Oligodendrocyte progenitor cells (OPCs) were regarded for many years solely in concern to their potential to generate oligodendrocytes and myelin in the central nervous system (CNS). However, accumulating data elucidate the multifaceted roles of OPCs, including their immunomodulatory functions, positioning them as cardinal constituents of the CNS’s immune landscape.

**Main body:**

In this review, we will discuss how the two therapeutic approaches converge. We present a model by which (1) an inflammation is required for the appropriate pro-myelinating immune function of OPCs in the chronically inflamed CNS, and (2) the immune function of OPCs is crucial for their ability to differentiate and promote remyelination. This model highlights the reciprocal interactions between OPCs’ pro-myelinating and immune-modulating functions. Additionally, we review the specific effects of anti- and pro-inflammatory interventions on OPCs, suggesting that immunosuppression adversely affects OPCs’ differentiation and immune functions.

**Conclusion:**

We suggest a multi-systemic therapeutic approach, which necessitates not a unidimensional focus but a harmonious balance between OPCs’ pro-myelinating and immune-modulatory functions.

## Background

Multiple sclerosis (MS) is an autoimmune neurodegenerative disease characterized by inflammation and progressive demyelination within the central nervous system (CNS) [1]. Therapeutic goals for MS have historically been segmented into two primary categories: strategies focusing on systemic-peripheral immunosuppression and those promoting pro-myelinating activity. This dichotomy has driven a significant amount of research and clinical efforts. Yet, neither approach has successfully provided a comprehensive therapy or a cure, especially for chronic progressive MS (CPMS), emphasizing the complex nature of the disease’s pathology.

Oligodendrocyte progenitor cells (OPCs), long recognized for their ability to generate mature myelinating oligodendrocytes, have traditionally been the focus of pro-regenerative strategies. The understanding of OPCs roles has evolved significantly in recent years. Emerging data illustrate the multifaceted roles of OPCs, moving beyond their myelin production capabilities to include substantial immunomodulatory functions [2], including response to inflammation-associated factors and dependence on them, expression of immune-related genes, antigen presentation, and secretion of immune-modulatory molecules. This paradigm shift repositions and suggests OPCs as pivotal players in the immune landscape of the CNS.

This review explores the intersection of immune-modulatory and pro-regenerative therapeutic approaches, suggesting a convergent model of these historically separate avenues. In addition, we review the specific effects of anti- and pro-inflammatory interventions on OPCs. We propose that inflammation is crucial to OPC pro-myelinating immune functions in the persistently inflamed CNS. Furthermore, we argue that these immune functions of the OPC itself are integral to its capacity to differentiate and promote remyelination. This hypothesis accentuates the intricate interplay between the pro-myelinating and immune-modulating functions of OPCs and highlights the necessity for a balanced, multi-systemic therapeutic approach.

## OPCs and myelin repair

### The pro-myelinating capabilities of OPCs

Oligodendrocytes, as highly specialized cells in the CNS, are chiefly recognized for their role in myelin synthesis. This multilamellar fatty membrane wraps around the axons to insulate them and facilitate efficient nerve impulse transmission [3, 4]. The intricate process of myelin formation involves the activation, migration, and differentiation of OPCs into mature myelinating oligodendrocytes [5, 6]. OPCs, characterized by their heterogeneity and multipotency, emerge during embryonic development and persist as resident cells in the adult brain parenchyma. Constituting approximately 6% of the total adult brain cell population [7, 8], OPCs are especially abundant in areas such as the subventricular zone (SVZ) and the corpus callosum [9].

A key part of remyelination is activation, which is stimulated in MS by demyelination. During demyelination, several chemoattractants, such as C–C motif chemokine ligand (CCL)2 and interleukin (IL)1, are released and promote the activation and migration of OPCs to the lesion site [10, 11]. The activation involves not only changes in morphology but also the upregulation of numerous genes involved in mature oligodendrocyte generation. These genes are encoded by many transcription factors, including Olig2, Nkx2.2, Myt1, and Sox2 [12–15]. The recruited OPCs then embark on a differentiation journey to transform into myelinating oligodendrocytes. This phase comprises three distinct steps: initial contact establishment with the demyelinated axon, myelin gene expression, myelin membrane generation, and ultimately, the wrapping and compacting of the membrane to form the myelin sheath.

While OPCs are capable of differentiating within active demyelinated lesions, their proliferation is inadequate to meet the repair needs in MS [16–18]. This insufficiency can partly be explained by the hostile environment in these lesions [17, 19, 20]. Consequently, the pool of OPCs in the lesion diminishes with each demyelination event, necessitating replenishment from OPCs in adjacent, unaffected tissues to sustain repair efforts.

### Promoting OPC differentiation as a therapeutic strategy for MS

The failure of remyelination is a cardinal impediment in CPMS and poses a formidable challenge for therapeutic intervention. CPMS is characterized by an impaired remyelination process due to various factors, including hindered migration of OPCs, OPC differentiation block, and failure in myelin formation around demyelinated axons despite successful differentiation [5, 6, 11, 21–26]. This understanding has redirected the search towards treatments that prevent neurodegeneration instead of just suppressing the immune system. This paradigm shift led to the study of various neuroprotective agents, pro-differentiation agents, or cell-based therapies [27–29].

Numerous studies have accentuated the inhibition of OPC differentiation into myelin-forming cells, and it has been posited that promoting OPC differentiation could potentially surmount remyelination failure in CPMS [30]. In light of this, much attention has been paid to identifying pathways and small molecules involved in OPC differentiation and remyelination. Multiple groups have screened for many small molecules in an attempt to find one that may change the course of CPMS [31–34]. For example, considering the role of protein-tyrosine phosphatase-α oligodendrocyte formation [35], studies have looked for PKC inhibitors [36], Rho inhibitors [37], ROCK-II activators [38], RXR agonists [39], MAPK/ERK inhibitor [34], and other small molecules that induce OPC differentiation for clinical use [40].

While MS is closely associated with remyelination failure, this issue also extends to other neurodegenerative disorders, including Alzheimer’s disease (AD). The cuprizone model, typically employed to study demyelination in MS, has also been adapted to explore AD pathology. Studies using this model reveal that cuprizone-induced demyelination not only leads to cognitive impairments but also causes significant changes in brain structure and function. For instance, mice treated with cuprizone exhibit deficits in learning tasks and show alterations in diffusion tensor imaging parameters, indicative of both demyelination and axonal damage [41]. These findings highlight the potential for mechanisms of OPCs differentiation and remyelination observed in MS to be applicable to other neurodegenerative diseases. Moreover, aging significantly impacts OPC function [42], with OPC senescence potentially leading to a reduction in many of their beneficial effects. Studying and understanding the molecular mechanisms and environmental factors that influence OPC differentiation can help identify common pathways and potential therapeutic targets that may be applicable across different neurodegenerative diseases.

Recently, there has been growing interest in the mechanisms by which dysregulated deoxyribonucleic acid (DNA) methylation patterns in MS hinder differentiation by activating Id2 and Id4 [43]. These two DNA-binding protein inhibitors typically act as negative regulators of differentiation under normal conditions. Another possible promising agent is metformin, which has been shown to decrease DNA damage and increase the metabolic function of OPCs in vivo [44]. Also, other studies underscore the significance of TMEM10 (Opalin) [45] or myelin regulatory factor (MYRF) [46] in enhancing OPC differentiation in MS. Another potential approach involves the use of antibodies to block Nogo to encourage differentiation and remyelination [47]. Additionally, Notch1 siRNA has been deployed to boost remyelination in a cuprizone model, as Notch1, while promoting OPC proliferation, also inhibits remyelination [48].

Research increasingly focuses also on enhancing OPC differentiation and proliferation through agents affecting lipid and cholesterol synthesis, which is vital for myelin structure, constituting 40% of its composition compared to 25% in typical cell membranes. Berghoff et al*.* [49] demonstrated that cholesterol supplementation promotes OPC proliferation and differentiation in neuronally active myelinating co-cultures. Similarly, Saher et al*.* [50] found that mice unable to synthesize cholesterol exhibited significantly reduced myelination, highlighting cholesterol’s crucial role. This understanding has spurred investigations into compounds like benztropine, a muscarinic antagonist that has shown efficacy in reducing clinical severity in both cuprizone and experimental autoimmune encephalomyelitis (EAE) models. The EAE model is particularly valuable as a standard approach to studying MS, utilizing induced autoimmune responses to simulate the disease. In a proteolipid protein (PLP)-induced EAE model of relapsing–remitting MS, benztropine enhanced remyelination, whether administered prophylactically or therapeutically at disease onset [33]. Selective estrogen receptor modulators (SERMs) such as tamoxifen also offer promise, promoting OPC differentiation via ERα, ERβ, or GPR30 and stimulating remyelination in vivo in models of focal demyelination [51, 52]. Moreover, high-throughput screenings have identified clobetasol and miconazole as effective remyelinating agents, promoting early myelination in organotypic cerebellar slice cultures and in vivo in neonatal mice [31]. Systemic delivery of these drugs also enhanced remyelination and improved outcomes in LPC-induced models and MOG35–55-induced EAE, which simulates chronic inflammatory demyelination [31]. Furthermore, OPCs express peroxisome proliferator-activated receptor (PPARs), and fatty acids, serving as endogenous ligands for these receptors, promote differentiation and antioxidant defenses of OPCs [53, 54]. Coupled with the knowledge that fatty acids can regulate inflammation [55], this may open an interesting avenue for research aimed at promoting remyelination.

Although none of these abovementioned potential agents have yet been explored in clinical trials, several therapeutic agents that have shown promise in animal models for promoting remyelination have progressed to phase 1 clinical trials. One such agent is the antibody rHIgM22, which not only enhances the clearance of myelin debris by microglia, but also stimulates the proliferation and remyelination of OPCs [56, 57]. However, current data do not attribute any immune-modulatory effects to it. Another promising compound is the aptamer LJM-3064, a 40-nucleotide single-stranded DNA sequence selected for its ability to bind to myelin. When conjugated with streptavidin and injected intraperitoneally into Theiler’s murine encephalomyelitis virus (TMEV) model mice, remyelination increased approximately four times compared to the control molecule, dT40 [58]. The results of these phase 1 clinical trials did not identify significant benefits from drugs known to promote oligodendrocyte differentiation [59]. One exception was the antimuscarinic drug clemastine fumarate, which showed a limited effect in reducing the latency of visual evoked potentials in eyes affected by chronic optic neuritis, suggesting improved remyelination [28]. Lastly, the use of induced pluripotent stem cells (IPSCs) and mesenchymal stem cells offers another promising pathway, as these cells have thus far produced encouraging results, albeit not yet in clinical settings [60–62].

The discrepancy between the promising approaches in preclinical studies and their clinical outcomes may stem from several unaddressed challenges. These include the widespread depletion of oligodendroglial populations in long-lasting MS and insufficient OPC recruitment to lesions, which can be hindered by the presence of migration-inhibiting molecules and the lack of migration-promoting factors [11, 26, 63]. Moreover, while these treatments predominantly target OPC differentiation, they do not address the complex interplay of environmental and cellular factors within MS lesions that are essential for successful remyelination [26]. This emphasizes the need for a more systemic approach that promotes OPC differentiation, replenishes oligodendroglial cells, and optimizes the lesion milieu to facilitate effective remyelination.

### The effects of currently approved therapies on OPC differentiation

The past quarter-century has witnessed remarkable progress in the development of MS treatments. Currently, more than 16 treatments for MS have been approved by the US Food and Drug Administration (FDA). All approved disease-modifying therapies (DMTs) target the systemic dysregulated immune responses, intending to restrain inflammation and prevent further myelin damage.

Although none of those treatments aimed to directly affect OPCs or glial cells, a couple of studies have explored DMTs’ impact on glial cells, specifically on OPC differentiation. First, interferon (IFN)-β, has been shown to attenuate demyelination in vivo, yet no significant effects on OPCs’ proliferation or differentiation have been reported in vitro [64, 65]. Moreover, it has been demonstrated to inhibit rat OPC differentiation in the presence of astrocytes and microglia [66]. Dimethyl fumarate has been observed to trigger OPC proliferation in vitro in a microglia-dependent manner, but its direct influence on OPCs remains unclear [67, 68]. Glatiramer acetate, primarily recognized for its impact on T-cell differentiation and activation [69], has demonstrated effectiveness by not only increasing the number of myelinated axons in EAE mice but also augmenting the mRNA expression of myelin basic protein (MBP) in EAE spinal cords [70, 71].

High-efficacy DMTs were also examined for their effect on OPCs. Fingolimod treatment has been shown to promote OPC differentiation in vitro [72], but it failed to enhance remyelination in the cuprizone-induced demyelination model [73]. Natalizumab was found to reduce demyelination in inflammation-mediated EAE. However, its direct impact on OPCs has yet to be examined [74]. Lastly, ocrelizumab, the only FDA-approved DMT for CPMS regardless of disease activity, has only been studied in relation to neurons and astrocytes, and its effects on oligodendroglial cells remain unknown.

## MS: a closer look at CNS inflammation

### Inflammation in MS

It is widely recognized that the immune system plays a central role in the pathogenesis of MS, with both the innate and adaptive immune systems contributing to the inflammation processes that lead to demyelination and axonal damage. The diagnostic hallmark of MS is the presence of inflammatory demyelinated lesions, primarily in the white matter of the CNS, with the destruction and loss of oligodendrocytes [75].

The inflammatory process in MS is characterized by the activation of T and B lymphocytes, macrophages, and microglia, as well as the activation of astrocytes during active tissue damage and the formation of gliotic scars in inactive lesions [1, 76, 77]. Thus far, the inflammatory milieu has been linked to the destruction of the myelin sheath, which is followed by axonal damage [78].

Given that MS is fundamentally a systemic autoimmune disorder, the primary therapeutic strategy has been designed to target peripheral immune cells and recalibrate the balance between systemic pro- and anti-inflammatory responses [79, 80]. Presently, approved DMTs vary in terms of efficacy, safety, and tolerability [81, 82]. Although various therapies employing different mechanisms of action are available for the relapsing forms of MS, therapies proven to be effective for CPMS are very limited [81, 82]. The effects of these treatments, particularly their inconclusive and limited impact on CNS resident cells, accentuate the need for alternative therapeutic strategies, especially for CPMS patients [83].

### The inflammatory process within the CNS

The understanding that peripheral immunosuppression fails in certain disease phases, along with the concept of compartmentalized pathogenesis of CPMS [75], illuminates the crucial role of the central immune system embodied by CNS-resident cells. It is widely accepted today that the brain’s innate immune system, traditionally represented by microglia, plays a paramount role in chronic neuroinflammation-induced brain injury [84]. Studies have indicated that different polarization states of microglia are responsible for different stages in EAE progression and repair processes. While the M1 phenotype, which is dominant following demyelination, contributes to OPC recruitment, a switch to the M2-dominant profile is essential to promote OPC differentiation [85, 86]. In LPC-induced demyelination models, both M1 and M2 phenotypes simultaneously play crucial roles in the demyelination-remyelination process [87]. Notably, a distinctive spatial distribution was observed in the LPC model, with M2 microglia localized within the core of the demyelination lesion, encircled by OPCs and oligodendrocytes, astrocytes, and M1 microglia [87]. This arrangement indicates that in addition to polarization, the spatial distribution of M1 and M2 phenotypes is a critical factor for OPC differentiation and the onset of remyelination. Interestingly, a switch from M1- to M2-associated gene expression has not been observed in studies of cuprizone-induced demyelination [88]. Nowadays, it is well established that the simplistic M1-M2 dichotomy does not reflect the highly complex nature of microglia, as these cells can polarize into a broad spectrum of phenotypes and activation states. One recently identified phenotype is the ‘microglia inflamed in MS’ (MIMS), which displays neurodegenerative programming in non-resolving MS lesions [89].

Astrocytes are the most abundant cell type in the CNS, and their involvement in immune responses is well-known as well: they express immune-related receptors [90], synthesize all components of the complement system, and produce both immunomodulatory and immunopathogenic cytokines and chemokines [91, 92]. Through releasing specific cytokines and chemokines, astrocytes can influence microglial activation and functionality [93].

Lastly, OPCs were regarded for many years solely in concern to their potential to generate oligodendrocytes and myelin in the CNS [3, 4]. However, the immune-modulatory role of OPCs in the CNS has gained considerable attention in recent years. It is now accepted that OPCs have immunomodulatory functions and may play a prominent role as part of the immune milieu of the CNS [2, 94].

### The immune capabilities of OPCs

Novel evidence has revealed that oligodendrocytes can acquire a disease-specific state characterized by the expression of immune-related genes [94–98]. It has been suggested that oligodendroglia are pre-programmed at the chromatin level and can rapidly activate an immune response in the context of disease and their surroundings [98]. A recent study utilizing IPSCs derived from skin biopsies of MS patients found increased immunological and inflammatory gene expression within oligodendrocyte lineage cells [99]. Evidence suggests that the inflammatory environment is important for OPCs’ immune functions [96, 100–102]. Jäkel and colleagues [95] utilized single-nucleus RNA sequencing and unveiled seven distinct clusters of oligodendrocytes and several additional OPC subclusters within the white matter of MS patients’ brains, including an immune-OPCs cluster. This indicates the potential relationship between oligodendrocyte heterogeneity and the varying functional states of these cells in MS progression.

Over the past decade, significant strides have been made in understanding the immune roles of OPCs. In the context of MS, OPCs have been demonstrated to possess the ability to internalize myelin debris via phagocytosis, a process mediated through the LRP-1 pathway [94, 103]. Although the in vivo significance of this function remains under investigation, it is speculated that by engulfing toxic debris, OPCs could enhance their immunomodulatory functions [104]. Moreover, OPCs exhibit the capability to express genes involved in antigen processing and presentation and can present antigens via the major histocompatibility complex (MHC) -I and -II [94, 105]. Beyond this, they can modulate T cell activation and proliferation [94, 97, 105, 106] and release cytokines and chemokines such as CCL2, CCL3, CCL5, CCL11, and tumor necrosis factor (TNF)α in response to injury [10, 97, 107]. Notably, these immune functions of OPCs have been shown to be intertwined with their pro-myelinating roles and to impact OPC differentiation [10, 97, 104, 107–111].

Furthermore, while not traditionally defined as an immune function, OPCs can modulate the immune response by interacting with the neurovascular unit, thereby regulating the infiltration of immune cells from the peripheral circulation. They have been implicated in the maintenance and repair of the blood–brain barrier (BBB) through the production of factors such as angiopoietin-1 (Ang1) and vascular endothelial growth factor (VEGF) [112, 113]. Also, juxtavascular and perivascular OPCs have been observed to accumulate near blood vessels in the EAE mouse model [114]. These factors promote the integrity of the endothelial cell layer and the tight junctions that comprise the BBB, thus helping to maintain an immune-privileged environment in the CNS.

It is important to note that in the context of CNS diseases, such as MS, stroke, epilepsy, or Alzheimer’s disease, OPCs have been shown to play both beneficial and detrimental immune-modulatory roles. In some cases, OPCs can promote tissue repair and neuroprotection by regulating inflammation, supporting remyelination, and secreting trophic factors [106, 115, 116]. However, in other instances, OPCs can contribute to disease progression by exacerbating inflammation, impairing BBB integrity, or failing to differentiate into mature oligodendrocytes [113, 117].

### Immune modulations within the CNS

The mounting evidence regarding the central immune system’s role in CPMS pathogenesis has prompted many groups to explore treatments that directly target the CNS, aiming to modulate its innate immune system.

A significant portion of this research has been directed at modulating microglia within the inflamed CNS. Goldfarb et al. [118] delved into the effects of electroconvulsive therapy (ECT) on microglial toxicity during chronic EAE. Their findings indicated that ECT not only halted the progression of clinical symptoms but also alleviated neuroinflammation, demyelination, and axonal damage. Pathological studies combined with ex vivo assays suggested that the therapeutic effect of ECT arose from reduced microglial toxicity without altering their phenotype [118].

Another avenue of exploration involves molecules targeting Bruton tyrosine kinase (BTK), a pivotal component of the B cell receptor signaling pathway [119]. Given the high BTK expression in microglia, these promising treatments potentially target two pivotal cell populations implicated in CPMS. One study revealed that treatment with a BTK inhibitor modified the activation of infiltrating myeloid cells and microglia, subsequently reducing axonal damage in the spinal cord during chronic progressive EAE [120]. A recent study demonstrated that in a model of toxic demyelination, BTK inhibition enhanced microglial clearance of myelin debris, thereby accelerating remyelination [121]. However, despite these promising findings, recent phase III trials presented at the ACTRIMS Forum 2024 revealed that BTK inhibitors, such as evobrutinib, did not meet the expected efficacy in reducing the annualized relapse rate compared to teriflunomide, nor did they demonstrate benefits on secondary endpoints [122, 123]. This emphasizes the challenges in translating preclinical successes to clinical efficacy.

Depletion of microglia with colony-stimulating factor 1 (CSF1) receptor antagonists is another investigated therapeutic approach. PLX5622, a CSF1 receptor antagonist, demonstrated variable results: while treatment of acute EAE reduced disease scores, suggesting a shift towards an anti-inflammatory microglial profile [124], its application in chronic EAE notably worsened disease progression and escalated mortality rates [125]. This aligns with reports that non-specific inhibition of microglia, including their beneficial functions, could be detrimental in disease settings [126]. Furthermore, another study found that while microglial depletion delayed the onset of EAE, it did not affect the final disease scores [127].

A recent genome-wide association study (GWAS) has highlighted several CNS genes, including DNM3, DYSF, and ZNF638, that are linked to disease progression and predominantly enriched in oligodendroglial cells [128]. These findings emphasize the importance of developing treatments that target CNS resident cells, particularly OPCs. To date, no CNS-targeted treatment has been approved for MS patients. Moreover, none of the potential therapies have assessed their impact on the immune functions of OPCs; instead, the focus has been solely on enhancing their pro-myelinating capabilities.

## Towards a comprehensive treatment: a multi-dimensional approach

**Fig. 1:**
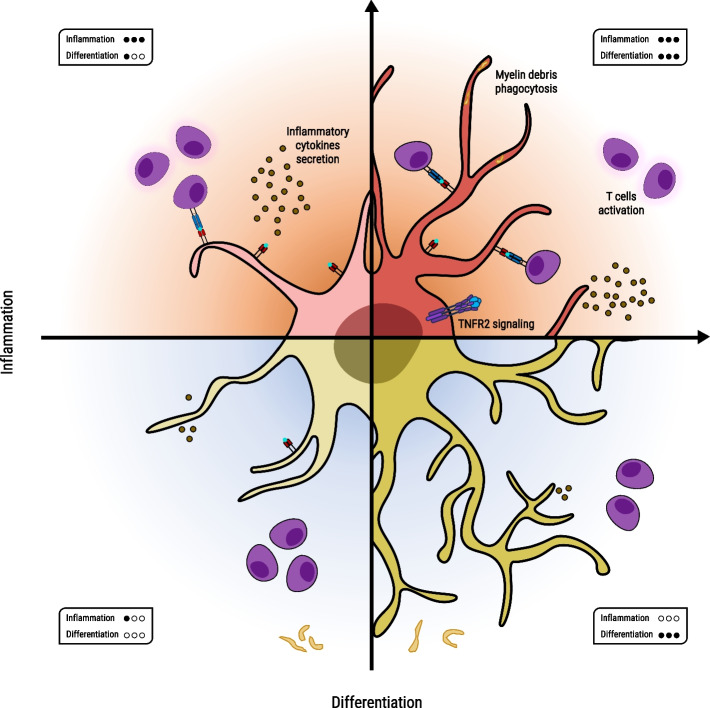
A multi-dimensional view: OPCs’ immune-modulation and pro-myelinating roles. Under inflammatory conditions, the CNS is abundant with detrimental inflammatory products such as IFNγ and myelin debris. These products suppress OPC differentiation while stimulating their immune activities, including phagocytosis, antigen presentation, and cytokine secretion [10, 104, 129, 130] (left upper panel). OPCs fail to differentiate into mature myelinating oligodendrocytes in environments with limited inflammation or anti-inflammatory conditions. Concurrently, their immune functions, such as antigen presentation, T-cell activation, and cytokine secretion, are compromised [97, 104, 131] (left lower panel). Pushing OPCs towards differentiation, combined with non-specific immune suppression, enables OPCs to differentiate into mature oligodendrocytes. However, this fails to ameliorate the disease burden in the *Biozzi* chronic EAE model (it also did not promote OPC migration to the areas of axonal loss). Additionally, their immune activities are dampened, preventing them from performing their immune-related roles [109, 132–134] (right lower panel). Successful remyelination relies on a well-regulated immune response, spatially and temporally controlled and of appropriate intensity [135, 136]. OPC must fulfill both roles: the regenerative and immune functions. OPCs are tasked with balancing both regenerative and immune functions. TNFα and oligodendroglial TNFR2 exemplify agents that harmonize the regenerative and immunological duties of OPCs within the inflamed CNS, endorsing both differentiation and immune-modulation [94, 97, 104, 111, 137] (right upper panel)

Here, we aim to discuss how the two therapeutic approaches converge. We suggest a multi-dimensional therapeutic approach, which necessitates a harmonious balance between OPCs’ pro-myelinating and immune-modulatory functions. Our intent is to shift the perspective from viewing inflammation and pro-myelination as independent entities towards recognizing that these dimensions intersect and coexist, as depicted in Fig. 1.

### Reduced differentiation and elevated inflammation

The concept that OPCs display immune functionality and actively participate in immune responses within the context of MS is relatively new. As a result, a limited pool of data is currently available on their immune-modulatory role and impact on the disease. Kirby et al*.* [105] studied postmortem MS brains and observed marked upregulation of the immunoproteasome subunit PSMB8 on oligodendrocyte lineage cells. Furthermore, their study revealed that OPCs can activate both CD4 + and CD8 + T cells, potentially leading to their own death in the inflamed CNS [105]. These findings suggest that such events might be pivotal in the chronic demyelination observed in MS patients.

In response to an assault, OPCs secrete cytokines and chemokines, such as CCL2, CCL3, CCL5, IL1β, and IL6, which might amplify neuroinflammation by attracting more peripheral and central immune cells, thereby accelerating neural damage [10, 129]. Notably, OPCs also react to CNS-present cytokines. For instance, IL6 and IL1β suppressed NG2 glial cell proliferation upon exposure to systemic LPS injections [138]. Additionally, OPCs were reported to disrupt the BBB, increasing its permeability to invading CD3 + lymphocytes and other peripheral immune cells [113, 139]. These may suggest that immune-activated OPCs may not only participate in MS pathogenesis but also instigate the initial event, aligning with the “inside-out” hypothesis.

The immune and pro-myelinating functions of OPCs are influenced by their environment, which can steer them towards different phenotypes. IFNγ is a key player in the immune response associated with MS [130, 140]. It is a type II interferon and is predominantly produced by activated T cells and natural killer (NK) cells. Notably, it is known for exacerbating the inflammatory response in MS. It promotes the activation and proliferation of immune cells, enhances the expression of MHC molecules, and facilitates the production of other pro-inflammatory cytokines, all of which contribute to the immune-mediated destruction of myelin in MS [130, 140].

IFNγ also affects OPCs. It activates OPCs and enhances their immune functions, such as antigen processing and presentation [97, 105] or nuclear factor kappa-B (NFκB) activation [97]. However, it is also known for its inhibitory effect on OPC differentiation [141, 142], and can even induce senescence in OPCs [143, 144]. Previous research using IPSCs indicated that blocking IFNγ could restore their capacity to differentiate into myelinating oligodendrocytes in the presence of an inflammatory environment [145]. In addition, Mezydlo et al*.* [146] recently demonstrated the cortical MS mouse model, which was achieved by double EAE induction followed by an intracortical injection of TNFα and IFNγ. They showed that surviving oligodendrocytes in the cortical MS mice model exerted inefficient remyelination, even under combined clemastine/metformin treatment, two agents that should stimulate remyelination [146]. IFNγ’s effects on OPCs’ immune and pro-myelinating capabilities highlight the necessity of further investigation into its complex role in MS pathogenesis. We previously assessed the effects of IFNγ on OPCs [104]. As expected, our experiments demonstrated its suppressive impact on OPCs’ differentiation and morphology. In addition, IFNγ was found to have a stimulatory role in activating the immune functions of OPCs, as evaluated by phagocytosis, MHC-II expression, NFκB activation, and the secretion of pro-inflammatory cytokines and chemokines [97, 104].

This simplified example demonstrates inflammatory scenarios that encourage OPCs’ immune activation but create an unfavorable environment for OPCs’ differentiation (Fig. 1, left upper panel). It suggests a rationale for the conventional belief that the prime therapeutic strategy for MS patients should focus on systemic immune-modulation.

### Reduced differentiation and reduced inflammation

In response to the necessity of systemic immune-modulation as a treatment strategy, we aim to delve into CPMS as a representative case study. This stage depicts a situation characterized by less inflammatory response compared to relapsing (rMS), yet it is devoid of OPC differentiation or remyelination.

While rMS is marked by the infiltration of peripheral immune cells and an increase in inflammatory proteins such as CXCL13, CXCL12, IFNγ, and TNFα [130, 140], the inflammation in CPMS is less systemic, being more contained and compartmentalized behind a relatively intact BBB [75, 147]. Numerous OPC differentiation regulators, like PDGF-AA, FGF2, IGF-I, TGF-β, and IL-1β, are heightened in acute inflammation and associated with the initial stages of lesion development [148–151], but are absent in a chronic inflammatory environment [6, 132]. Moreover, chronic lesions generally contain a low density of OPCs, potentially due to the presence of OPC repellents, such as myelin debris, within the lesions, which impair OPC recruitment and differentiaion or slow it down [26, 63, 152–156]. Consequently, OPCs often arrive at the axons after the inflammatory stimuli have subsided, and the axons have become non-permissive for remyelination [26]. As a result, oligodendrocyte repopulation and subsequent remyelination appear to function well in active demyelinating lesions but not in chronic MS lesions [16].

Our prior work demonstrated that the cerebrospinal fluid (CSF) of CPMS patients reduced the differentiation and immune functions of OPCs, including MHC-II expression, cytokines secretion, and T-cell activation and proliferation, compared to the CSF of rMS patients where these functions remained intact [97]. We also observed a lower immunological transcriptome in OPCs cultured with CSF of CPMS patients compared to rMS patients [97]. These findings were further supported by a recent in vivo study that demonstrated motor disability and spinal cord pathology, including demyelination, impaired remyelination, and axonal damage following the injection of CSF from primary progressive MS patients into the subarachnoid space of mice [131]. Additionally, another work demonstrated that IPSCs from peripheral blood mononuclear cells (PBMCs) derived from CPMS donors exhibit lower efficiency in differentiating into mature oligodendrocytes than those from rMS and HC donors [62]. Given that PBMCs inherently carry an immune signature, this observation suggests a primed linkage between differentiation and immune function.

Previous studies have suggested a pro-regenerative effect of anti-inflammatory environments. For instance, intranasal delivery of IL4 nanoparticles robustly promoted OPC differentiation and improved long-term functional recovery after stroke [157]. Furthermore, treatment of OPCs with conditioned media generated from microglia exposed to IL13 or IL10 but not IFNγ and LPS significantly enhanced oligodendrocyte differentiation [116]. A recent study demonstrated that extracellular vesicles derived from IL4-stimulated macrophages enhanced OPC maturation in preclinical models of MS [158]. A pivotal note is that the current data provide indirect insights and have been gathered under diverse conditions, which could mask a direct understanding of the effects on OPCs. These findings prompted us to examine the direct impact of anti-inflammatory mediators (IL4 and IL10) on OPCs in a previous study [104]. We found that exposure to IL4 and IL10 hindered OPC differentiation and reduced their immune functions, as shown by decreased phagocytosis, MHC-II expression, and pro-inflammatory cytokine secretion. Further research is essential to fully understand the influence of these mediators on OPCs in MS.

Our observations, coupled with earlier findings regarding the need for a permissive pro-inflammatory environment for successful remyelination, suggest that the pro-inflammatory milieu may also activate the immune functions of OPCs. This activation could facilitate remyelination through various activities, such as antigen presentation and cytokine secretion, which promote the migration and differentiation of OPCs.

Employing CPMS as a case study illuminates a scenario characterized by a reduced peripheral inflammatory environment and compartmentalized inflammation, where neither differentiation nor remyelination occurs (Fig. 1, left lower panel). This scenario underlines why therapeutic strategies concentrated purely on immune-modulation have not achieved the anticipated outcomes, reinforcing the notion that this system is far more intricate than a simple binary switch.

### Elevated differentiation and reduced inflammation

Considering that immunosuppression alone doesn’t appear to induce pro-differentiation effects, the next concept explored is the stimulation of OPCs to differentiate, thereby fostering regeneration in MS patients. This hypothesis postulates that a single agent capable of both promoting differentiation and concurrently suppressing inflammation could be the game-changer in treating MS (Fig. 1, right lower panel).

Numerous research groups have undertaken extensive screenings of various small molecules, aiming to identify a potential compound that could induce remyelination and potentially alter the course of CPMS [31–34]. Suo et al*.* [34] investigated the impact of PD0325901, a MAPK/ERK inhibitor, on the acute EAE model and the cuprizone-induced demyelination model. PD0325901 has been previously reported to cause robust and non-specific immune suppression, affecting different cell populations [159]. Therefore, it might be an ideal candidate for this purpose. Indeed, PD0325901 induced OPC differentiation in vitro and demonstrated a significant therapeutic effect in acute demyelination MS models [34].

These compelling results encouraged us to examine the effects of PD0325901 on the chronically inflamed CNS [109]. Treatment with PD0325901 induced OPC differentiation into mature oligodendrocytes with high morphological complexity both in vitro and in vivo. However, PD0325901 treatment of *Biozzi* mice with chronic-progressive EAE did not result in any clinical improvement compared to the control group, nor did it reduce demyelination or stimulate OPC migration into demyelination foci. PD0325901 had a broad immunosuppressive effect on multiple cell populations, resulting in reduced phagocytic capability of microglia and less activation of lymph node cells. It also significantly impeded the immune-modulatory functions of OPCs, as determined by their ability to activate lymph node cells and to secrete cytokines [109].

As discussed above, numerous small molecules and antibodies aimed at enhancing OPC differentiation have been tested and have shown encouraging results in animal models. However, as of yet, none have been advanced into clinical use [160]. This is supported by earlier findings that adequate remyelination requires not only a favorable environment, including pro-regenerative inflammatory elements, T cells, macrophages, and cytokines [116, 132–134, 161–163], but also effective OPC migration, repopulation, and differentiation [26, 152, 153, 164]. Importantly, this pro-regenerative inflammatory environment activates the immune functions of OPCs, enabling them to operate synergistically and may promote successful remyelination.

The failure to promote clinical improvement using a singular agent, which simultaneously encourages OPC differentiation while suppressing their immune functions, underscores that these dual roles cannot be addressed independently. Instead, they should be approached as interconnected components of a cohesive whole. This highlights the need for a multi-systemic therapeutic approach, which necessitates not a unidimensional focus but a harmonious balance between OPCs’ pro-myelinating and immune-modulation functions. Such an approach might hinge on identifying beneficial immune mediators and employing combinations of agents for optimized outcomes.

### Elevated differentiation and elevated inflammation

Accumulating evidence over the past few years illuminates the delicate equilibrium between inflammation and regeneration, indicating the need for more complex strategies to foster remyelination, especially in CPMS. We suggest an approach that simultaneously stimulates OPCs’ immune function and promotes OPC differentiation (Fig. 1, right upper panel).

Although historically, inflammation in the CNS was regarded as a destructive process, recent discoveries have shown that, like other regenerative processes, successful remyelination is associated with inflammation. For example, research has shown that in models of spinal cord injuries, both effector and regulatory T cells play crucial roles in facilitating tissue repair [165–167]. This view is further supported by earlier observations highlighting that OPCs and ongoing remyelination are found in active inflammatory MS lesions but rarely in immunologically inactive plaques [168–171]. Furthermore, in animal models of chronic demyelination, effective remyelination in OPCs was only achieved upon the induction of acute inflammation [132, 133, 172]. Behi et al*.* [102] revealed that a pro-inflammatory environment results in increased OPC differentiation through crosstalk with microglial cells. Their observations also discerned heterogeneity in the remyelination pattern in MS patients; high remyelination ability was found in correlation to microglial activation and lymphocyte cytokine secretion [102, 173]. Also, OPC cultures with Th1 cell supernatants led to increased differentiation [174, 175]. For example, IL1 can enhance the differentiation of OPCs and promote the maturation and survival of differentiating oligodendrocytes [176].

Further evidence proposes that the inflammation process contributes both to the myelin damage and repair processes [177]. While the pathology of MS is primarily immune-mediated, the innate immune response to demyelination creates a conducive environment for remyelination [178]. Immune cells play a crucial role in clearing myelin debris, which contains proteins that inhibit OPC differentiation [38, 156, 179–183]. Significantly, macrophage function in MS extends beyond debris clearance; their lipid metabolism also plays a critical role in remyelination and may substantially influence successful remyelination by OPCs [184, 185]. However, it is essential to note that cholesterol for remyelination also at least partially originates from de novo synthesis by oligodendrocytes [184, 186, 187]. Additionally, steroid administration, exemplifying non-specific immunosuppression, was found to delay CNS remyelination in vivo [188]. These are further validated by prior research, which has indicated that experimental depletion of macrophages [134], and B or T cells [161, 189] leads to remyelination impairment. We previously demonstrated that the inflammatory milieu in the CNS affects the immune and regenerative capabilities of OPCs, highlightinig the complex interplay between inflammation and remyelination [97, 104, 109].

Another study, through direct lineage analysis, reported accelerated remyelination following the induction of EAE [190]. They proposed the idea that the newly generated myelin maintains its stability at the peak of inflammation. Our previous work demonstrated that OPCs exposed to CSF of rMS patients had higher capabilities of immune functions and differentiation compared to those exposed to CSF of CPMS patients (Fig. 1, right upper panel) [97]. OPCs cultured with CSF from rMS patients expressed an upregulated immune-like transcriptome. They also demonstrated enhanced immune capacities, including MHC-II expression, NFκB activation, cytokine secretion, and T-cell activation. These are vital functions that might allow OPCs to maintain a regenerative environment in the CNS. Such functions can guide other OPCs or immune cells to the lesion site to clean myelin debris, initiate neuroprotective signaling pathways, aid the immune system in controlling inflammation, and ultimately foster remyelination [10, 129, 191]. Additionally, a recent study has shown that OPCs expressing MHC-I, correlated with areas of high inflammation, also exhibit elevated levels of PDL-1/CD274 [110]. Presumably, this helps to prevent CD8-mediated destruction, thereby enabling these cells to facilitate tissue repair. These observations substantiate the notion that for effective CNS repair, OPCs need to perform their dual roles—pro-myelinating and immune-modulating functions.

TNFα, a multifaceted cytokine, is abundantly present in the serum, CSF, and active lesions of MS patients [192, 193]. TNFα was observed to enhance both the differentiation and immune functions of OPCs [104]. Intriguingly, when OPCs were exposed to TNFα and IFNγ simultaneously, differentiation levels matched those exposed to TNFα alone and were significantly higher than those exposed to IFNγ alone [104] (Fig. 1, right upper panel). TNFα signaling can occur through two receptors: TNFR1, which mainly promotes neurotoxicity, and TNFR2, which fosters neuroprotection and reparative effects [194]. Recent studies spotlighted the essential role of oligodendroglial TNFR2 in modulating the inflammatory response following demyelination. EAE mice lacking oligodendroglial TNFR2 exhibited earlier microglial activation, peripheral immune cell infiltration, increased demyelination, widespread axonal loss, and hampered remyelination compared to their wild-type counterparts [111]. Gene expression profiling further revealed that the absence of oligodendroglial TNFR2 led to a substantial upregulation of various inflammatory mediators, in contrast to naïve mice [111]. This implies that oligodendroglial TNFR2 activation may help suppress the production of inflammatory signals, thereby limiting excessive neuroinflammation and reducing demyelination rates.

Intriguingly, in vitro experiments propose that TNFR2 plays a more prominent role in modulating the inflammatory response in OPCs compared to mature oligodendrocytes [137]. TNFR2 limits the pro-inflammatory phenotype of OPCs, and its absence exacerbates the immunomodulatory and inflammatory function of OPCs following inflammatory stimulation (by IL1β, IFNγ, and TNFα), diminishing their capacity to proliferate and differentiate. A recent breakthrough from Fiedler et al*.* [195] demonstrated the benefits of co-modulating TNFR1 and TNFR2 in an EAE model, with the result of effectively ameliorating the symptoms of EAE, as well as decreased demyelination, inflammatory infiltration, and axonal degeneration. The combined approach of inhibiting TNFR1 while stimulating TNFR2 signaling enhanced the survival rate of retinal ganglion cells and promoted the phosphorylation of both Akt and NFκB, both known to mediate neuroprotection [195]. In line with these, in vitro neutralization of TNFR2 resulted in reduced levels of differentiation, unaffected MHC-II expression, and elevated cytokine secretion [104]. These findings highlight the crucial role of TNFα and TNFR2 in striking a balance between the regenerative and immunological functions of OPCs in the inflamed CNS. Furthermore, the critical role of TNFα in preserving a regenerative environment within the CNS is further backed by cases where patients who were administered anti-TNF medications developed demyelinating syndromes [196].

Highlighting the prospects of immune-modulatory approaches aimed at regeneration, Genchi and colleagues [197] recently published the outcomes of a phase 1 clinical trial where they intrathecally injected neuronal precursor cells (NPCs) into patients with progressive MS. The premise of the trial was that NPCs could provide trophic support and immunomodulation, paving the way for neuroprotection and tissue repair. This represents a significantly distinct strategy compared to the anti-inflammatory compounds previously tested in CPMS. While the trial did not yield changes in clinical activity or disease progression, magnetic resonance imaging (MRI) analyses revealed an effect on the reduction of gray matter volume. Strikingly, in half of the participating patients, new lesions were identified, an unexpected development in a population with advanced, progressive disability (expanded disability status scale; EDSS 7). The emergence of inflammatory activity was particularly surprising given the concurrent use of tacrolimus to prevent rejection of the transplanted NPCs. Furthermore, the researchers performed an extensive set of CSF analyses, which indicated an up-regulation of trophic factors and immune-related molecules, as well as cytokines and chemokines [197]. These encouraging results hint at the potential of a therapeutic approach that fosters neuroprotection and combines regenerative strategies with the creation of a carefully regulated, inflammatory, and permissive environment.

These underline the importance of inflammation in MS and illuminate the burgeoning understanding that inflammation in MS might not always be detrimental. Therefore, remyelination is contingent on a well-regulated immune response, spatially and temporally controlled and of appropriate intensity, though the precise mechanisms remain elusive (Fig. 1, right upper panel) [135, 136]. Understanding the interplay between immune function and remyelination may help in devising new strategies for promoting OPC repopulation and differentiation as pro-remyelination therapies in MS.

## Conclusion

The body of knowledge gathered over the years elucidates the intricate relationship between inflammation and regeneration in the context of CPMS. The commonly held view that inflammation solely contributes to CNS damage and MS progression might be overly simplistic. Instead, certain elements of inflammation could be harnessed to stimulate regeneration, particularly by influencing the differentiation and immune functions of OPCs.

The growing body of evidence that positions OPCs as active contributors to the immune landscape of the CNS supports the necessity for a balanced inflammatory environment, aiding the adequate function of OPCs. Executing their essential immune roles would enable them to promote remyelination [10, 97, 107, 109, 111, 197].

The failure of single-dimensional immunosuppression-focused therapies illuminates the complex dynamics between inflammation and regeneration. For instance, the impact of the MAPK/ERK inhibitor on chronic progressive EAE elucidates that a fine balance must be struck between fostering a pro-regenerative environment and controlling destructive inflammation [109].

Simultaneously, we highlight the existence of specific pro-inflammatory environments that can potentially enhance OPC differentiation and consequent remyelination. The role of the multifaceted cytokine TNFα and the specific involvement of TNFR2 are prime examples of this complex interplay (Fig. 1) [104, 111, 137]. These factors can stimulate OPC differentiation and immune functions, adding more layers to our understanding of the inflammation-regeneration relationship.

By understanding the inflammation-regeneration relationship in MS, we can gain insights that may apply to other neurodegenerative diseases and vice versa. This cross-disease approach could lead to the development of more effective treatments for a range of conditions characterized by remyelination failure.

We present a model wherein inflammation is required for the appropriate immune function of OPCs in the chronically inflamed CNS. Furthermore, this immune function of OPCs is critical for their capability to differentiate and stimulate remyelination. This model accentuates the interconnected nature of OPCs’ pro-myelinating and immune-modulatory roles. While further studies are required to ascertain how and to what extent this equilibrium should be maintained, we advocate for a shift in the treatment paradigm for MS. We propose a multi-systemic therapeutic approach, which necessitates not a unidimensional focus but a harmonious balance between OPCs’ pro-myelinating and immune-modulation functions.

## Data Availability

Not applicable.

## Abbreviations

AD: Alzheimer’s disease
Ang1: Angiopoietin-1
BTK: Bruton tyrosine kinase
CNS: Central nervous system
CCL: C-C motif chemokine ligand
CPMS: Chronic progressive multiple sclerosis
CSF1: Colony-stimulating factor 1
CSF: Cerebrospinal fluid
DNA: Deoxyribonucleic acid
DMTs: Disease-modifying therapies
EDSS: Expanded disability status scale
EAE: Experimental autoimmune encephalomyelitis
FGF: Fibroblast growth factor
FDA: Food and Drug Administration
GWAS: Genome-wide association study
IFN: Interferon
IL: Interleukin
IPSCs: Induced pluripotent stem cells
MHC: Major histocompatibility complex
MIMS: Microglia inflamed in MS
MAPK/ERK: Mitogen-activated protein kinase/extracellular signal-regulated kinase
MS: Multiple sclerosis
MBP: Myelin basic protein
MYRF: Myelin regulatory factor
NK: Natural killer
NPCs: Neuronal precursor cells
NFκB: Nuclear factor kappa-B
OPCs: Oligodendrocyte progenitor cells
PBMCs: Peripheral blood mononuclear cells
PKC: Protein kinase C
PTPα: Protein-tyrosine phosphatase-α
rMS: Relapsing multiple sclerosis
ROCK-II: Rho-associated protein kinase
SVZ: Subventricular zone
TMEV: Theiler’s murine encephalomyelitis virus
TGF: Transforming growth factor
TMEM: Transmembrane protein
TNF: Tumor necrosis factor
TNFR: Tumor necrosis factor receptor
VEGF: Vascular endothelial growth factor
VEP: Visual evoked potentials
